# The effect of nutritional factors on urolithiasis: A case-control study

**DOI:** 10.25122/jml-2022-0321

**Published:** 2023-07

**Authors:** Ehsan Shabani, Ali Khorshidi, Kourosh Sayehmiri, Kamran Moradi, Ehsan Nabi Abdolyousefi

**Affiliations:** 1 Epidemiology Student Research Committee, Faculty of Health, Ilam University of Medical Sciences, Ilam, Iran; 2 School of Medicine, Ilam University of Medical Sciences, Ilam, Iran; 3 Psychosocial Injuries Research Centre, Ilam University of Medical Sciences, Ilam, Iran; 4 Department of Urology, Faculty of Medicine, Ilam University of Medical Sciences, Ilam, Iran.; 5 Genetic Student Research Center, Faculty of Medicine, Shiraz University of Medical Sciences, Shiraz, Iran

**Keywords:** nutritional factors, urolithiasis, urinary tract stones

## Abstract

Urolithiasis, a prevalent chronic kidney disease affecting all age groups, can be influenced by nutritional factors. The incidence of urolithiasis in Asian countries ranges from 1% to 19.1%, attributed to geographical and lifestyle differences. In Iran, several risk factors, including ethnicity, dietary habits, gender, and age, are associated with urolithiasis. This study aimed to assess the impact of nutritional factors on kidney and urinary tract stone formation. This case-control study enrolled 127 newly diagnosed urolithiasis patients, and 127 matched healthy participants between February to May 2017. Exclusion criteria included diabetes and acute or chronic renal failure. Data were collected using the Food Frequency Questionnaire (FFQ) and analyzed using chi-square and logistic regression tests. Water (95% CI: 0.09-0.89, OR=0.28), natural juices (95% CI: 0.10-0.65, OR=0.53), mineral water (95% CI: 0.05-0.64, OR=0.18), legumes (95% CI: 0.00-0.38, OR=0.032), butter, cream, or peppermint (95% CI: 0.09-0.95, OR=0.30), and ice cream (95% CI: 0.07-0.54, OR=0.203) had a significant protective effect against kidney and urinary tract stone formation. Conversely, tea consumption (95% CI: 1.15-7.99, OR=4.70), beverages (95% CI: 4.45-23.32, OR=23.32), coffee (95% CI: 1.63-11.78, OR=4.39), bread (95% CI: 1.1-10.59, OR=3.37), meat (95% CI: 1.01-8.01, OR=2.85), liver (95% CI: 3.37-488.90, OR=40.58), fish (95% CI: 2.89-216.39, OR=25.03), and various canned foods (95% CI: 1.34-10.25, OR=3.70) were significantly associated with kidney and urinary tract stone risk. These findings showed that the risk of urinary stones formation had a significant relationship with dietary habits. Therefore, the correct dietary pattern and sufficient fluid consumption may play an important role in preventing urinary stones.

## INTRODUCTION

Renal stone disease, also known as urolithiasis, is a prevalent chronic condition affecting individuals of all ages [1]. The incidence is 5 to 13 percent in men and 4 to 7 percent in women [2, 3]. Urinary tract stones are an important health problem with a remarkable share in surgeries and kidney failure patients [4]. The annual rate of stone formation in industrialized countries ranges from 1,500 to 2,000 per million individuals, with approximately 25% of patients requiring active stone removal [5]. In a study conducted in Iran in 2005, the prevalence of the disease was reported to be 7.5% [6]. Recurrence of calcium stones is also observed in around 60% of patients within 10 years [7].

The association of urinary stones with diet is somewhat known, but there are controversial cases in this area. For example, while the shortage of protein intake is one of the major causes of endemic rocks in developing countries such as Iran, India, and Turkey, high protein consumption is one of the most important factors in the formation of stones in industrialized countries. It is believed that there is no reason to justify an increase in protein intake and a higher incidence of urinary stones [8]. In the past, patients with calcium kidney stones were advised to limit calcium intake; however, current evidence questions the validity of this issue [9]. Numerous nutritional factors can alter urinary compositions and cause over-saturation, and, as a result, affect the process of stone formation [10]. Too much protein, sodium, calcium, and oxalate sources intake may increase the risk of stone formation in susceptible individuals [11]. Dietary risk factors have a different effect on the formation of urinary stones based on age and sex. It has been reported that calcium, phytate, and sufficient fluid intake are associated with reduced risk of urinary stones in young women, while animal proteins and sucrose are associated with increased risk. In older adults, calcium intake does not have any effect with age increase, but magnesium, potassium, and fluids intake are associated with a reduced risk of developing urinary stones, and vitamin C increases the risk of symptomatic stomachs [12]. Although the role of consuming adequate fluids in preventing the formation and recurrence of urinary tract stones has been reported in limited studies, some studies mentioned that fluid intake in some kidney disease patients exacerbates the homeostasis of the body [13-15]. The best way to control the disease and its complications, especially in developing countries, is to prevent the growth of stones or the formation of new stones [16].

Based on our observations in society, we recognized the need to study the underlying factors contributing to urolithiasis. This study focuses on urolithiasis in Ilam, a province in Iran with a higher prevalence than other provinces. Additionally, meat consumption is relatively higher in Ilam, raising questions about its potential association with urolithiasis. Our study aims to promote awareness among healthcare professionals and the general public about the frequency of fluid and food consumption to prevent kidney and urinary tract stone formation. We also aim to inform educational programs to increase patients' awareness of urolithiasis and its causes to prevent and reduce recurrence.

Given these considerations, health education focusing on dietary and lifestyle modifications for individuals with kidney and urinary tract stones holds promise. In this study, we primarily investigate the association between nutritional factors and the formation of kidney and urinary tract stones, guided by input from health experts. Importantly, there is a dearth of research on the epidemiology of urinary stones, and the fluid intake and diet patterns in urolithiasis patients remain unclear. Furthermore, there is limited information on the preventive effects of fluid consumption on urinary stone formation. Therefore, this study aimed to determine the effect of nutritional factors on the formation of kidney and urinary tract stones in Northeast Iran in 2017.

## BACKGROUND

### Study design and setting

This case-control study was conducted in Ilam health centers from February to May 2017. A total of 127 patients with urolithiasis and 127 control subjects without kidney stones were included in the study. The control group was selected to match the urinary tract characteristics of the case group. Individuals with diabetes, acute or chronic renal failure, and those on dialysis or under special care were excluded.

### Participants

The sample size was calculated using the Cochran formula with a 0.05 error and a 95% confidence interval (1.96) [17]. Patients were recruited from kidney and urinary tract clinics in Ilam City. Age and sex were used as matching criteria to ensure comparability between the case and control groups. The same matching criteria were applied to the control group. Patients were included if they were diagnosed with urinary stones based on ultrasound scans conducted at specialized clinics. Control group subjects were selected from individuals without urinary stones according to their ultrasound examination at specialized clinics. Patients with acute or chronic renal failure, diabetes, dialysis, or people under special care were excluded.

### Clinical assessment and data collection

Demographic and disease characteristics were collected for both groups. Physical activity levels were assessed using the Baecke Physical Activity Questionnaire (BQ) [18]. Data were collected through direct interviews conducted by trained experts. The dietary intake pattern was assessed using the Food Frequency Questionnaire (FFQ), which was validated and proven reliable in previous studies [19, 20]. The frequency of consumption was determined for all types of market bread, local bread, meat, rice, liver, chicken and poultry, all types of canned food, fish, eggs, legumes (beans, lentils, etc.), sausages, salami, and burgers, salad, greens, nuts, and dried fruits, all kinds of fruits, milk, whey, yogurt, cheese, butter, cream, snacks, chocolate, all types of cakes and pastries, sugar, tomato, ice cream, shrimp, carbonated beverages, beverages, dough, coffee, natural juices, industrial juices, minerals, water, and tea. Response options ranged from never to rarely, daily, and weekly.

### Statistical analysis

Statistical analysis was conducted to assess the relationship between the independent variables and the dependent variable. Binary logistic regression was employed to predict the effect of the independent variables on urolithiasis. Additionally, data were analyzed using the chi-square test and logistic regression model. The analysis was performed using SPSS 20 software, following the necessary data exploration and modeling steps. The dependent variable of interest was the presence of urolithiasis, while the independent variable comprised various nutritional factors. These included types of market bread, local bread, meat, rice, liver, chicken and poultry, all types of canned food, fish, eggs, legumes (beans, lentils, etc.), sausages, salami, and burgers, salad, greens, nuts and dried fruits, all kinds of fruits, milk, whey, yogurt, cheese, butter, cream, snacks, chocolate, all types of cakes and pastries, sugar, tomato, ice cream, shrimp, carbonated beverages, beverages, dough, coffee, natural juices, industrial juices, minerals, water, and tea.

## RESULTS

### Demographic characteristics

In this study, 254 subjects participated, with 127 subjects included in the case group and 127 in the control group. 180 subjects (70.9%) were male, and 74 (29.1%) were female. The two groups matched regarding age and sex. The demographic characteristics of patients with kidney stones and healthy subjects are shown in Table 1. According to the demographic information, Body Mass Index (BMI) was statistically different between the two groups (p=0.001). The main water consumption in this study was plumbing water, but there is no data about the composition of plumbing water in this city.

**Table 1:** Demographic characteristics of participants

Variable	Case (n=127)	Control (n=127)	Sig.
**Age** <40 40-60 60<	63 45 19	63 45 19	1.00
**Gender** Male Female	90 37	90 37	1.00
**Occupation** Worker Employee Others	19 34 74	19 37 71	.91
**Education** Illiterate Low literacy Diploma and university	19 26 82	12 31 84	.36
**Marital status** Married Single	104 23	98 29	.35
**Place of residence** Urban Rural	99 28	100 27	.87
**Economic status** Low Medium High	19 78 30	23 76 28	.788
**Body mass index** Slim and normal Overweight Obese	48 63 16	81 37 9	.001
**Daily activity** No activity 30 minutes One hour More than an hour	23 35 15 54	25 32 22 48	.594
**History of urinary stones in first-degree relatives** Yes No	69 58	63 64	.451

Chi-square test was used to analyze the data. The analysis steps were performed using SPSS 20 software.

### Fluid-related factors

The effect of fluid intake and dietary factors on urolithiasis was examined using logistic regression models. The analysis included both crude and adjusted models, controlling for baseline variables such as age, body mass index, physical activity level, geographic region, specific health profession, use of thiazide diuretics, alcohol intake, drinking sugar cola, coffee intake, and dietary intake of calcium, animal protein, sucrose, magnesium, sodium, phosphorus, potassium, vitamin D, and total fluid (Table 2). The type of kidney stones observed in the study was primarily calcium oxalate, with an average size of 10.33±6.31 mm, and the stones were mostly located in the inferior calyx. Variables concerning the frequency of consuming water, tea, soda, natural juices, industrial juices, dough, mineral water, and coffee were entered into the model. Regarding water intake, individuals consuming 4-5 glasses of water daily had a 72% lower risk of kidney and urinary tract stones than those consuming up to one glass (OR=0.28, 95% CI: 0.09-0.89). On the other hand, individuals consuming 4-5 glasses (OR=3.63, 95% CI: 1.15-13.84) or at least 6 glasses (OR=4.70, 95% CI: 1.15-17.99) of tea daily had a higher risk of kidney and urinary tract stones compared to those consuming up to one glass a day.

**Table 2:** Relationship between fluid intake and the risk of developing kidney and urinary tract stones

Variable	Case (n=127)	Control (n=127)	Crude OR (95% CI)	Adjusted OR (95% CI)
**Water** One glass per day> 2-3glasses per day 4-5glasses per day At least 6 glasses per day	23 41 34 29	8 30 55 34	1	1
			0.47(0.18, 1.20) 0.21(0.08, 0.53)^*^ 0.29(0.11, 0.76)^*^	0.48(0.15, 1.58) 0.28(0.09, 0.89)^*^ 0.39(0.11, 1.32)
**Tea** One glass per day> 2-3 glasses per day 4-5 glasses per day At least 6 glasses per day	7 36 39 45	17 51 32 27	1	1
			1.71(0.64, 4.55) 2.96(1.09, 8.01)^*^ 4.04(1.48, 11.01)^*^	1.85(0.50, 6.84) 3.63(1.15, 13.84)^*^ 4.70(1.15, 17.99)^*^
**Soft drink** Never Rarely Weekly Daily	37 45 27 18	42 58 24 3	1	1
			0.88(0.48, 1.58) 1.27(0.63, 2.58) 6.81(1.85, 24.98)^*^	0.97(0.43, 2.20) 2.50(1.01, 6.40)^*^ 23.32(4.45, 23.32)^*^
**Natural juice** Never Rarely Weekly Daily	43 40 38 6	16 49 57 5	1	1
			0.30(0.14, 0.61)^*^ 0.24(0.122, 0.50)^*^ 0.44(0.11, 1.66)	0.21(0.09, 0.54)^*^ 0.53(0.10, 0.65)^*^ 0.45(0.07, 2.85)
**Industrial juice** Never Rarely Weekly and daily	55 36 36	21 65 41	1	1
			0.21(0.11, 0.40)^*^ 0.33(0.17, 0.65)^*^	0.19(0.08, 0.44)^*^ 0.18(0.07, 0.46)^*^
**Dough** Never Rarely Weekly Daily	12 34 48 33	5 18 70 34	1	1
			0.78(0.24, 2.58) 0.28(0.09, 0.86)^*^ 0.40(0.12, 1.27)	1.88(0.37, 9.40) 0.48(0.11, 2.04) 0.93(0.20, 4.27)
**Mineral water** Never Rarely Weekly Daily	49 39 28 11	24 48 26 29	1	1
			0.39(0.20, 0.75)^*^ 0.52(0.25, 1.87) 0.18(0.08, 0.43)^*^	0.59(0.26, 1.33) 0.97(0.37, 2.52) 0.18(0.05, 0.64)^*^
**Coffee** Never Rarely Weekly and daily	65 30 32	83 29 15	1	1
			1.32(0.72, 2.41) 2.72(1.36, 5.45)^*^	2.13(1.98, 4.65)^*^ 4.39(1.63, 11.78)^*^

Chi-square test and logistic regression model were used to analyze the data. The analysis steps were performed using SPSS 20 software.

The consumption of soft drinks or syrup of less than 2 glasses per day did not show a significant effect on the reference level (never). However, weekly and daily consumption of soft drinks significantly increased the odds ratio of developing kidney and urinary tract stones (daily: OR=23.32, 95% CI: 4.45-23.32; weekly: OR=2.50, 95% CI: 1.01-6.40). Weekly and rare consumption of natural juices showed a significant protective effect against kidney and urinary tract stones (weekly: OR=0.53, 95% CI: 0.10-0.65; rare: OR=0.21, 95% CI: 0.09-0.54). Similarly, weekly and rare consumption of industrial juices was associated with a lower risk of kidney and urinary tract stones compared to never consuming them (weekly: OR=0.18, 95% CI: 0.07-0.46; rare: OR=0.19, 95% CI: 0.08-0.44).

Daily consumption of mineral water compared to the reference level (never) as a significant protective factor reduced the odds ratio of kidney and urinary stones up to 82% (95% CI: 0.05-0.64, OR=0.18). On the other hand, weekly and daily consumption of coffee increased the odds ratio of developing kidney and urinary tract stones (weekly: OR=4.39, 95% CI: 1.63-11.78; daily: OR=2.13, 95% CI: 0.98-4.65). The variables were categorized into four groups: never (<1 glass per day), rarely (2-3 glasses per day), weekly (4-5 glasses per day), and daily (at least 6 glasses per day).

### Dietary-related factors

A logistic regression model was employed to investigate the association between diet type and the risk of urolithiasis, as shown in Table 3. The frequency of consuming various food items such as market bread, meat, liver, fish, various canned foods, beans, butter, cream, cereal, and ice cream was included in the model.

**Table 3:** The relationship between the frequency of dietary intake type and the risk of developing kidney and urinary tract stones

Variable	Case (n=127)	Control (n=127)	Crude OR (95% CI)	Adjusted OR (95% CI)
**Market bread types** Never Rarely Weekly Daily	7 9 7 75	22 16 14 104	1	1
			1.76(0.54, 5.74) 1.57(0.45, 5.45) 4.35(1.77, 10.72)^*^	1.90(0.43, 8.32) 1.01(0.21, 4.78) 3.37(1.01, 10.59)^*^
**Meat** Rarely Weekly Daily	19 67 41	34 76 17	1	1
			1.57(0.82, 3.02) 4.31(1.94, 9.57)^*^	1.29(0.56, 2.97) 2.85(1.01, 8.01) ^*^
**Liver consumption** Never Rarely Weekly Daily	18 80 21 8	35 69 22 1	1	1
			1.85(0.81, 4.23) 2.25(1.17, 4.33)^*^ 15.55(1.80, 14.33)^*^	2.12(0.73, 6.18) 3.50(1.49, 8.26)^*^ 40.58(3.37, 488.90)^*^
**Fish** Never Rarely Weekly Daily	6 70 43 8	30 59 36 2	1	1
			5.93(2.31, 15.22) 5.97(2.23, 15.94)^*^ 20.00(3.37, 5.22)^*^	7.19(2.12, 24.39)^*^ 13.50(3.71, 49.10)^*^ 25.03(2.89, 216.39)^*^
**Canned food types** Never Rarely Weekly	38 54 35	48 66 13	1	1
			1.03(0.59, 1.80) 3.40(1.58, 7.31)^*^	0.89(0.43, 1.88) 3.70(1.34, 10.25)^*^
**Legume** Never Rarely Weekly Daily	9 28 83 7	2 13 96 16	1	1
			0.47(0.09, 2.53) 0.19(0.04, 0.91)^*^ 0.09(0.01, 0.57)^*^	0.25(0.02, 2.59) 0.10(0.01, 0.93)^*^ 0.032(0.00, 0.38)^*^
**Butter, cream or kaymak** Never Rarely Weekly Daily	25 38 46 18	12 22 55 38	1	1
			0.82(0.34, 1.97) 0.40(0.18, 0.88)^*^ 0.22(0.09, 0.55)^*^	0.955(0.32, 2.86) 0.57(0.21, 1.60) 0.30(0.09, 0.95)^*^
**Ice-cream** Never Rarely Weekly and daily	41 48 38	18 57 52	1	1
			0.37(0.18, 0.72)^*^ 0.032 (0.16, 0.64)^*^	0.233(0. 090, 0.605)^*^ 0.203(0.176, 0.544)^*^

Chi-square test and logistic regression model were used to analyze the data. The analysis steps were performed using SPSS 20 software.

Table 3 demonstrates the relationship between dietary factors and the risk of developing kidney and urinary tract stones. Daily consumption of market bread showed a significant difference compared to the reference level (never), with individuals who never consumed market bread being more likely to develop kidney and urinary tract stones (OR=3.37, 95% CI: 1.01-10.59). Regarding meat consumption, individuals who consumed meat daily had a higher likelihood of developing stones compared to those who consumed meat rarely (OR=2.85, 95% CI: 1.01-8.01). Similarly, daily (OR=40.58, 95% CI: 3.37-488.90) and weekly (OR=3.50, 95% CI: 1.49-8.26) liver consumption increased the odds of kidney and urinary tract stones compared to never consuming liver.

The odds ratios for kidney and urinary tract stones were higher in individuals who consumed fish daily (OR=25.03, 95% CI: 2.89-216.39), weekly (OR=13.50, 95% CI: 3.71-49.10), and rarely (OR=7.19, 95% CI: 2.12-24.39) compared to those who never consumed fish. Weekly consumption of canned food also had a significant effect, with individuals who consumed canned foods weekly being more likely to develop kidney and urinary tract stones than those who never consumed them (OR=3.70, 95% CI: 1.34-10.25).

Daily and weekly consumption of legumes had a significant effect on the reference level (never) so that the odds ratios of individuals consuming legumes weekly (95% CI: 0.01-0.93, OR=0.10) or daily (95% CI: 0.00-0.38, OR=0.032) were less likely to have kidney and urinary tract stones than people who never eat legumes.

The consumption of butter and cream exhibited a significant protective effect, reducing the risk of kidney and urinary tract stones. Individuals who consumed cream and butter daily had a 70% lower risk of developing these stones than those who never consumed them (95% CI: 0.09-0.95, OR=0.30). Similarly, when examining ice cream consumption, the odds ratios for daily and weekly intake were 0.203 (95% CI: 0.176-0.544, OR=0.203), indicating a decreased likelihood of kidney stones and urinary tract issues. Even rare consumption of ice cream showed a reduced risk, with an odds ratio of 0.233 (95% CI: 0.090-0.605, OR=0.233), compared to individuals who never consumed it.

## DISCUSSION

According to the findings of this study, fluid consumption had a significant relationship with the formation of kidney and urinary tract stones. Increasing fluid intake reduces the concentration of compounds that can lead to deposition in the urine. On the other hand, it reduces the free crystalline particles in the urine [21]. There is also evidence that adequate fluid intake can prevent the recurrence of urinary stones [13, 14, 22]. This study indicated that people who drank four to five glasses of water a day were less likely to have urinary stones than those who drank one glass of water a day, consistent with the results of Sorensen *et al*. [23]. In his study, Anderson showed that the risk of recurrence of stones in people with minimum water consumption is 41% higher compared to people with maximum water consumption [4].

Diet can change urine chemistry and affect stone formation. Studies have reported varying calcium and oxalate levels in patients, with some showing high levels and others showing low levels [24-27]. In conclusion, calcium and oxalate are risk factors connected with stone formation and urinary stones [28-31]. Studies showed that calcium can increase the rate of excretion and absorption and bind to oxalate in the gut [30, 31].

Previous research has demonstrated that consuming cola and carbonated beverages can lead to increased oxalate secretion, which contributes to the formation of calcium oxalate stones [22]. Demographic studies in Iran have identified the consumption of carbonated beverages and beverages as risk factors for urinary stones. In addition, tea consumption is also known as a risk factor [32]. Anderson's research findings indicated that consuming cola-containing drinks, which are high in phosphorus content, could potentially contribute to the formation of calcium oxalate stones [16]. The study also found that increased tea consumption (4-5 glasses per day) and daily and weekly consumption of soft drinks increased the chances of developing kidney and urinary tract stones. The findings of this study showed that the usual consumption of liquids such as natural juices, industrial juices, and minerals decreased the risk of kidney and urinary tract stones, and this is consistent with the results of another study reporting that a fluid-rich diet reduces the risk of developing urinary tract stones [33].

Moreover, other studies indicated that mineral water containing calcium and magnesium could serve as a preventive and therapeutic agent for kidney and urinary tract stones [34, 35]. Aras *et al*. also found that the consumption of lemon juice could be a potential treatment for patients with urinary calcium stones [36], supporting the findings of the current study. In a meta-analysis by Wang *et al*., coffee consumption reduced the risk of renal and urinary tract stones [37]. This was inconsistent with the present study revealing that increased coffee intake enhanced the risk of developing renal and urinary tract stones. In a study conducted by Borghi *et al*., increasing fluid intake to 2 liters of urine resulted in a significant reduction in calcium and oxalate concentrations and reduced the rate of recurrence of stones [38].

It is widely acknowledged that increasing fluid intake is beneficial for patients with urinary stones. However, limited information is available regarding the specific effects of different beverages on the formation of urinary stones [39]. This study showed that coffee consumption was high in our study sample, and drinking coffee was more common in patients with stones. In addition, there was a significant difference in tea intake. Some studies reported that drinking coffee and alcoholic beverages reduce the risk of stones, and consuming beverages containing bicarbonate increases the risk of associated stone formation [39, 40].

The findings regarding tea consumption are also inconsistent. In Curhan's study, consuming various types of tea, coffee, and alcoholic beverages (with or without caffeine) was associated with a reduced risk of urinary stones, while no relationship was found between the consumption of bicarbonate beverages and the risk of urinary stones [39]. The heterogeneity in our study compared to other studies may be attributed to differences in the frequency of fluid consumption based on drinking habits and lifestyle in the respective locations.

According to our findings, dietary intake was significantly correlated with the formation of urolithiasis. Daily consumption of various types of market bread increased the odds ratios of urinary stones, which aligns with the results reported by Trinchieri [41]. However, Khani *et al*. found no significant correlation between bread consumption and kidney and urinary tract stones, which is inconsistent with the present study [42]. Furthermore, our study revealed that daily meat consumption increases the risk of kidney and urinary tract stones, consistent with the findings of Nguyen *et al*. [43], who observed a direct correlation between meat protein intake and kidney stones.

Liver consumption had a significant relationship with the risk of developing renal and urinary tract stones, although this relationship has not been directly mentioned in previous studies. However, Neun *et al*. reported that the consumption of animal protein can increase the risk [44]. Chard *et al*. observed a significant relationship between fish consumption and the risk of renal stone formation [45], consistent with our findings. Additionally, our study revealed that the consumption of canned foods was significantly associated with the risk of kidney and urinary tract stone formation, with weekly consumption increasing the risk, in line with Lekcharoensuk *et al*. [46].

Our study demonstrated that regular consumption of legumes reduced the risk of developing kidney and urinary tract stones. Our findings contradict the study by Dai *et al*. [47], which observed a positive relationship between legume consumption and kidney stone formation in women. The disparity may be due to differences in food consumption patterns, lifestyle, and the larger number of healthy individuals in our study. Similarly, our study showed that daily consumption of butter, cream, or kaymak decreased the odds ratio of urolithiasis, contrasting with the results of Khani *et al*. [42]. The discrepancy may be attributed to variations in food consumption patterns, lifestyle, and the larger number of healthy individuals in our study.

Furthermore, our study revealed that the consumption of ice cream reduced the chance of developing kidney urolithiasis. This finding contrasts with the findings of Khani *et al*. [42], who did not find a significant correlation between ice cream consumption and urinary stones in their research.

The heterogeneity in findings across these studies may be attributed to differences in food consumption frequencies and lifestyle habits among the study populations. It is important to note that diet plays a significant role in determining urine chemistry and can influence the risk of stone formation. Numerous studies have explored different diets and their lithogenic and prophylactic effects, particularly in calcium oxalate urolithiasis. Among the diets studied, those with high and low amounts of calcium and oxalate, either separately or combined, have been the most extensively examined [24-27]. Several excellent reviews analyzed the impact of dietary calcium and oxalate on the urinary risk factors linked to the formation of calcium oxalate stones [28-31]. These studies have provided valuable insights into the relationship between dietary and urinary risk factors.

Evidence from these studies suggests that calcium restriction can improve oxalate absorption and excretion while increasing calcium intake can bind more oxalate in the intestine and reduce its presence in the urine. This mechanism is supported by two large prospective studies showing a reduced risk of stone formation with increased dietary calcium intake [30, 31]. On the other hand, other studies have demonstrated a direct correlation between dietary oxalate and oxalate excretion [28].

In our study, the statistical population was divided into healthy individuals and patients, excluding other groups of patients with conditions such as acute or chronic renal failure, diabetes, dialysis patients, or those under special care. In a study by Nouvenne *et al*. in 2014, the effect of unhealthy eating habits on the formation of urinary stones was high. Urinary factors, such as high protein diets and significant urinary citrate levels in patients, were identified as important risk factors for kidney stone formation [44].

Our study had some limitations, such as the length of the Food Frequency Questionnaire (FFQ), which was time-consuming and sometimes led to incomplete responses from patients [38, 39]. Additionally, there were challenges related to patient recall of information, limitations in data collection, non-cooperation from some participants, and the inclusion of elderly individuals.

## CONCLUSION

The findings of this study emphasize the significant impact of dietary factors on the occurrence of kidney and urinary tract stones. It is evident that careful adherence to specific dietary patterns can substantially reduce the prevalence of these conditions.

## Data Availability

The data for this study is available upon request.

## Personal thanks

The authors thank the Vice-Chancellor of Research at Ilam University of Medical Sciences and everyone who helped us in this project.
